# Predictors of survival among children and adolescents with rhabdomyosarcoma treated in a single resource-limited centre —Uganda

**DOI:** 10.1186/s12885-025-14735-3

**Published:** 2025-08-11

**Authors:** Richard Nyeko, Fadhil Geriga, Racheal Angom, Joyce Balagadde Kambugu, Jaques van Heerden

**Affiliations:** 1 Department of Paediatrics and Child Health, Lira University, P.O. Box 1035, Lira, Uganda; 2 Division of Paediatric Oncology, Uganda Cancer Institute, P.O. Box 3935, Kampala, Uganda; 3 Department of Paediatric Haemato-Oncology, Antwerp University Hospital, University of Antwerp, Antwerp, Belgium

**Keywords:** Rhabdomyosarcoma, Children, Adolescents, Outcomes, Resource-limited contexts

## Abstract

**Background:**

The treatment outcomes for children and adolescents with rhabdomyosarcoma (RMS) in low-income countries are poor. However, there is a paucity of literature on RMS and its management outcomes in low-resource settings. We evaluated the treatment of RMS with the aim of identifying prognostic factors during management to improve outcomes.

**Methods:**

We sourced data on children under 18 years treated for rhabdomyosarcoma at the Uganda Cancer Institute between January 2016 and December 2020. Kaplan-Meier survival analysis and Cox’s proportional hazards model were used for five-year survival analysis.

**Results:**

One hundred twenty-eight RMS cases were identified, with a median age of 6.0 years (IQR 3.6–10.0). The most common primary tumour site was the head and neck region, comprising non-parameingeal sites, 37 (28.9%); parameingeal sites, 32 (25.0%); and orbital tumours, 17 (13.3%). Overall, 68 (53.1%) of the primary tumour sites were unfavourable sites. Seventeen (13.3%) patients had metastatic disease at diagnosis, primarily to the lungs, 11 (64.8%). Embryonal and alveolar RMS accounted for 50.0% and 20.3% of the cases, respectively. Only 31 (24.2%) of the patients underwent surgery, and 36 (28.1%) were irradiated. The treatment completion rate was 33.6%, while 46.1% abandoned treatment. Only 25 (19.5%) patients were alive at the time of the study, 65 (50.8%) had died, and 38 (29.7%) had an unknown status. The five-year overall and event-free survival rates were 35% and 30%, respectively. Orbital primary tumour site (HR = 2.86; 95% CI 1.12–7.31; *p* = 0.028), metastatic disease (HR = 4.09; 95% CI 2.01–8.31; *p* < 0.001), elevated serum lactate dehydrogenase at diagnosis above 400 U/L (HR = 2.80; 95% CI 1.46–5.33; *p* = 0.002), and lack of local control (HR = 3.33; 95% CI 1.34–8.29; *p* = 0.010) were significant factors for poor survival.

**Conclusion:**

Rhabdomyosarcoma outcomes in Ugandan children are largely poor, with high treatment abandonment and mortality. Concerted, multidisciplinary efforts are needed to improve outcomes in this setting.

**Supplementary Information:**

The online version contains supplementary material available at 10.1186/s12885-025-14735-3.

## Background

Rhabdomyosarcoma (RMS) is the most common soft tissue sarcoma in children, with an incidence of 4.5/one million children [1]. It accounts for approximately 3–7% of all childhood cancers [2–4] and more than 50% of all soft tissue sarcomas in children and adolescents [2, 5]. In Uganda, RMS accounts for approximately 4% of all paediatric cancer [6].

Over the past decades, the use of a multimodal therapeutic approach involving a combination of multi-agent therapy and effective local control using surgery (primary or secondary) and/or radiotherapy [2, 4, 7] has improved the cure rates of RMS from a dismal 25% in the 1970 s to a rate over 70% [8–11]. In high-income countries (HICs), patients with non-metastatic, low-risk RMS reach over 90% survival [12]. Even in the face of the advances in therapy, about 30% of children with RMS experience poor outcomes characterised by disease progression, relapse, and death [4, 8].

The treatment of childhood RMS remains exceptionally challenging in LICs despite the improved outcome in HICs. Survival in children with RMS in developing countries has remained in the region of 10%−45% [4, 13]. This is occasioned by the need for multidisciplinary management setups [2, 14] that may not be available in many LIC centres. Delay in diagnosis and advanced disease stage, treatment abandonment and relapse, and failure to provide adequate local control in resource-limited settings may contribute to poor outcomes in children with RMS in LICs [2, 15–17]. Thus, survival has varied across geographical and clinical settings depending on various prognostic factors unique to various resource settings.

The current data on RMS treatment come largely from published literature of patients treated on cooperative group trials in HICs, where several clinical and biological variables with proven or possible prognostic significance have been evaluated [1, 17, 18–20]. Nonetheless, the relative contribution and significance of some of these prognostic factors have not been evaluated in LICs, where other patient-, disease-, treatment-related, and context-specific factors may influence outcomes. This study evaluated the predictors of survival among children and adolescents with rhabdomyosarcoma treated in a single resource-limited Ugandan centre.

## Methods

### Study design and setting

**Fig. 1:**
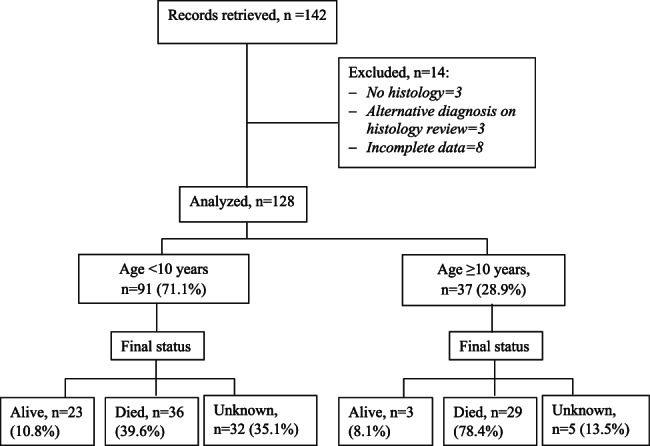
Study Flow diagram

This study was retrospective, and records of children and adolescents aged below 18 years diagnosed with rhabdomyosarcoma were reviewed. The sample included patients treated at the Uganda Cancer Institute (UCI) between January 2016 and December 2020. The UCI is Uganda’s only national reference cancer treatment centre, treating nearly 80% of the children with cancer in the country. Patients with an uncertain or inconclusive diagnosis, incomplete medical records lacking clinical details, or an alternative diagnosis on histology review were excluded (see Fig. 1).

### Study procedure and data extraction

Demographic information (age and sex/gender), duration of symptoms, tumour characteristics and stage of disease, basic laboratory investigations (complete blood counts and serum lactate dehydrogenase [LDH]), patient management, and clinical course and outcome were collected. Where the participant’s status could not be ascertained owing to a lack of documentation or lost-to-follow-up, a phone call follow-up was made to the caregiver to enquire and ascertain the child’s status.

### Rhabdomyosarcoma diagnosis and treatment

The diagnosis of RMS was made on the basis of clinical presentations and radiological findings. This was confirmed by histological examination of tissue biopsy based on the morphologic criteria defined by the World Health Organization (WHO) classification [21]. Other basic laboratory investigations, such as complete blood count and serum lactate dehydrogenase levels, among other tests, were also performed. However, molecular, cytogenetic, and biologic studies are not available at the UCI. The staging workup included radiological evaluations with a computed tomography (CT) scan or plain X-ray, ultrasonography, and bone marrow trephine examination. Pre-surgical clinical staging was assigned according to the Intergroup Rhabdomyosarcoma Study Group (IRSG) pre-surgical staging classification [9]. Clinical grouping was assigned according to the IRSG postsurgical grouping classification.

In the study setting, children with RMS were managed with multimodal therapy according to a local protocol based on risk stratification. Patients in the low-intermediate group received vincristine, doxorubicin, and cyclophosphamide (VDC) alternating with vincristine, actinomycin D, and cyclophosphamide (VAC) every 3 weeks (see Additional file 1), while the high-risk groups received VDC alternating with ifosfamide, vincristine, and actinomycin D (IVA) every 3 weeks, all along with vincristine every week in the intervening period between two three-drug cycles for the first 12 weeks. Patients also received mesna with cyclophosphamide and ifosfamide to prevent haemorrhagic cystitis (see Additional file 2). Local control consisted of surgery, radiation therapy, or both. Surgical excision of the primary tumour was considered after at least 4 cycles of neoadjuvant chemotherapy, followed by radiotherapy to the primary site using conventional fractions– unless the patient had upfront surgery, where adjuvant chemotherapy and radiotherapy would be given. Interval radiological evaluations were performed prior to surgery, and individuals with resectable tumours were offered surgery. Where surgical excision was not feasible, like in the case of head and neck/para-meningeal RMS, radiotherapy followed neoadjuvant chemotherapy. Physical examination, radiographic investigations, and biopsy, where feasible and appropriate, were used to confirm recurrences (local or systemic). In the absence of a national health insurance, and with the exception of chemotherapy and laboratory investigations, patients have to foot the costs of other aspects of their treatment, including radiological investigation, radiotherapy, transportation costs, upkeep and other expenses.

### Clinical and outcome definitions

Tumour size was taken as the maximal tumour diameter, defined as the largest radiologically estimated diameter of the tumour. Severe acute malnutrition was defined as a weight-for-height/length z-score less than − 3 or mid-upper-arm circumference < 11.5 cm for children six months to 59 months and a body mass index-for-age z-score less than − 3 for children five to 17 years. Moderate acute malnutrition was defined as a weight-for-height/length z-score ≥ −3 but less than − 2 or mid-upper-arm circumference ≥ 11.5 cm but less than 12.5 cm for children six months to 59 months and a body mass index-for-age z-score ≥ −3 but less than − 2 for children five to 17 years. An LDH level cut-off of 400 U/L was chosen based on the prognostic level documented by Weng-Ling et al. [22]. We defined delayed presentation (patient delayed diagnosis) as the time interval from the onset of symptoms and signs to presentation at the cancer treatment facility of three months or more. Presentation before three months was considered very early, while that between 3 and 6 months was considered early. Overall survival (OS) was defined as the time duration from the date of diagnosis to death from any cause. Event-free survival (EFS) was defined as the time duration from the date of diagnosis to the first event (disease progression, relapse, or death from any cause, whichever came first). Treatment abandonment was defined as failure to initiate or complete treatment. This excluded the decision of palliative treatment or discontinued treatment due to toxicity by primary oncologists.

### Statistical analysis

Data were analysed using the Statistical Package for Social Sciences (SPSS) software package (SPSS for Windows, Version 20.0. Chicago, SPSS Inc.). Descriptive statistics were summarised as proportions for categorical variables, while continuous variables were summarised as means (standard deviation) if normally distributed or median (interquartile range) if non-normally distributed. Survival analysis was estimated using the Kaplan-Meier method and compared using the log-rank test [23]. Patients alive at the end of the period under consideration for survival analysis or at the last follow-up date were censored. The effect of covariates on survival was estimated using Cox proportional hazards analysis [24]. The variables found to be statistically significant and those with a *p*-value < 0.2 in the univariate analysis were included in the multivariate model. Hazard ratios (aHR) were generated with the associated 95% confidence intervals (CI). A two-sided *p*-value < 0.05 was considered for statistical significance.

## Results

### Description of the study participants

Data from 128 children and adolescents diagnosed with rhabdomyosarcoma were analysed (Fig. 1).

The median age at diagnosis was 6.0 years (interquartile range 3.6–10.0), ranging from one to 16 years, with the majority, 91 (71.1%), of the participants aged less than 10 years. More than half, 70 (54.7%), were males, and the majority, 80 (62.5%), presented with symptoms lasting three months or more. Forty-nine (38.5%) of the participants had B symptoms, and 41 (32.0%) were malnourished—23 of whom (56.1%) had moderate acute malnutrition (MAM) while 18 (43.9%) had severe acute malnutrition (SAM) (Table 1).

**Table 1:** Demographic and clinical characteristics among children and adolescents with rhabdomyosarcoma (*n* = 128)

**Variable **	**n**	**%**
Age (years)		
<10	91	71.1
≥10	37	28.9
Sex		
Male	70	54.7
Female	58	45.3
Duration of symptoms (months)		
˂3	48	37.5
3-6	54	42.2
˃6	26	20.3
B symptoms		
Yes	49	38.3
No	79	61.7
Specific B symptoms		
Fever	34	26.6
Weight loss of more than 10%^a^	35	27.3
Drenching night sweats	27	21.1
Nutritional status		
Normal	80	62.5
Malnourished	41	32.0
Unknown	7	5.5
Degree of malnutrition (*n*=41)		
MAM	23	56.1
SAM	18	43.9
Site of primary tumour		
Head and neck– Non parameningeal	37	28.9
Head and neck - Parameningeal	32	25.0
Orbital	17	13.3
Genitourinary (bladder & others)^c^	14	10.9
Extremity	12	9.4
Abdomen	10	7.8
Other^b^	6	4.7
Prognostic site		
Favourable	60	46.9
Unfavourable	68	53.1
Size of primary tumour		
≤5cm	31	24.2
>5cm	71	55.5
Unknown^d^	26	20.3
Lymph mode involvement		
N1	54	42.2
N0	69	53.9
Nx	5	3.9
Tumour invasiveness		
T1	48	37.5
T2	69	53.9
Tx	11	8.6
Group		
I	0	0.0
II	2	1.6
III	109	85.2
IV	17	13.3
Distance metastasis at diagnosis		
M1	17	13.3
M0	88	68.7
Mx	23	18.0
Site of metastasis		
Lung	11	64.8
Liver	3	17.6
Bone/Bone marrow	3	17.6
Histological subtypes		
Embryonal/Botryoid	64	50.0
Alveolar	26	20.3
Pleomorphic	11	8.6
Not otherwise specified	27	21.1
Risk stratification		
Low risk	23	18.0
Intermediate risk	71	55.5
High risk	25	19.5
Unknown	9	7.0

*Abbreviations:* *IQR* interquartile range, *MAM *Moderate acute malnutrition, *SAM *Severe acute malnutrition

^a^Weight loss of 10% was either derived from a patient’s clinical history or objectively measured comparative to a previous measurement

^b^Peritoneum, pelvis, and thorax; Nx, Tx and Mx=not evaluated

^c^bladder (8), paratesticular (2), vagina, vulva, uterus, kidney (1 each). T1 Tumour confined to anatomic site of origin (non-invasive); T2 Tumour extension and/or infiltration of surrounding tissues (invasive)

^d^radiological reports had no estimated tumour size

The most common primary tumour site was the head and neck, comprising non-parameningeal sites, 37 (28.9%) of the cases; parameningeal sites, 32 (25.0%) of the cases; and the orbit, 17 (13.3%) of the cases. Over half, 68 (53.1%), of the primary sites were unfavourable prognostic sites. Seventy-one (55.5%) of the primary tumour lesions measured more than 5 cm in the longest diameter at diagnosis (median 8.0 cm [IQR 5.0–12.0]), and 53.9% were locally invasive (T2)– i.e., tumours extending and/or infiltrating surrounding tissues. The majority, 109 (85.2%), of the patients were in IRS group III, and metastatic disease (IRS group IV) at the time of diagnosis was evident in 17 (13.3%) of the patients, 11 (64.8%) of whom had pulmonary metastases, and three (17.6%) each had liver and bone/bone marrow metastases, respectively. The most common histological subtype was embryonal/botryoid in 64 (50%) of the patients, while alveolar and pleomorphic subtypes accounted for 20.3% and 18.6%, respectively. A substantial proportion of histological diagnoses, 27 (21.1%), were not otherwise specified (Table 1).

**Fig. 2:**
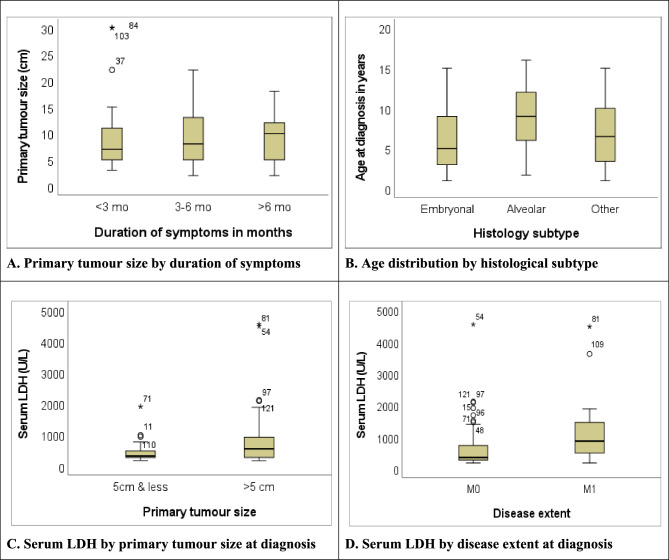
Box plots of clinical, histopathological characteristics, and serum LDH level. (**A**) Primary tumour size by duration of symptoms. (**B**) Age distribution by histological subtype. (**C**) Serum LDH by primary tumour size at diagnosis. (**D**) Serum LDH by disease extent at diagnosis

Primary tumour size was larger for children with symptoms lasting more than six months (Fig. 2A). Embryonal RMS occurred mainly in younger children (median age 5.5 years [IQR 3.0-9.3]), and alveolar RMS was more common in older children (median age 9.0 years [IQR 6.0–12.0]) (Fig. 2B).

### The laboratory parameters

The median serum albumin and haemoglobin values at diagnosis were 39.0 mg/dL (range 20.0-51.1) and 11.1 g/dL (range 3.2–14.9), respectively. The median serum LDH value at diagnosis was 427.75 U/L (range 180–4549), and that of ALP was 196.0 U/L (range 67.0-1831.0) (Table 2). The serum LDH was higher for patients with primary tumour size measuring more than 5 cm in the longest diameter (Fig. 2C) and in patients with metastatic disease at diagnosis (Fig. 2D).

**Table 2:** Laboratory parameters, treatment characteristics and clinical outcomes of the children <18 years with rhabdomyosarcoma (*n*=128)

**Laboratory parameters**		
Variable	Median (IQR)	Range
Albumin (mg/dL)	39.0 (33.0-42.6)	20.0-51.1
Haemoglobin (g/dL)	11.1 (9.5-12.5)	3.2-14.9
ALP (U/L)	196.0 (127.4-256.7)	67.0-1831.0
LDH (U/L)	427.75 (274.3-834.8)	180 - 4549
**Treatment characteristics and clinical outcomes**		
Variable	n	%
Chemotherapy		
Yes	120	93.7
No	8	6.3
Time from first visit to therapy		
≤3 weeks	78	67.8
˃3 weeks	37	32.2
Local control		
Yes	57	44.5
No	71	55.5
Surgery		
Yes	31	24.2
No	97	75.8
Timing of surgery		
Upfront	16	51.6
Pre-surgical chemotherapy	15	48.4
Extent of surgery/surgical margins		
R0	4	12.9
R1	8	25.8
R2	7	22.6
Unknown	12	38.7
Surgical resection by prognostic site^a^		
Favourable site	20	33.3
Unfavourable site	11	16.2
Radiation therapy		
Yes	36	28.1
No	92	71.9
Treatment progress		
Completed treatment	43	33.6
Abandoned treatment	59	46.1
Others^b^	26	20.3
Final outcome		
Alive	25	19.5
Died	65	50.8
Unknown	38	29.7

*LDH* Lactate dehydrogenase, *ALP* Alkaline phosphatase, *R0* Complete resection/negative margins, *R1* Microscopic residual disease, *R2* Macroscopic residual

^a^only the percentage of patients in each prognostic site who had surgery are indicated, hence not totalling to 100%

^b^Incomplete treatment due to disease progression, palliation, and mortality

### Treatment characteristics and clinical outcomes

The majority, 120 (93.7%), of the patients received chemotherapy as one of the modalities of treatment, typically within three weeks or less (median 13.0 weeks [IQR 6.0–27.0]) from the time of the first visit to the UCI. Local control was performed in less than half, 57 (44.5%), of the participants. Nearly one quarter, 31 (24.2%), underwent surgical tumour resection, of which 16 (51.6%) were upfront surgeries and 15 (48.4%) followed preoperative courses of chemotherapy. Only twelve (38.7%) of these resections were complete, with negative margins in four and positive margins in eight. In another 12 (38.7%) of the cases, the degree of resection could not be ascertained. Thirty-six (28.1%) of the patients received radiotherapy. Only one of the seven patients with incomplete tumour resection received radiotherapy. In general, only 43 (33.6%) of the patients completed the prescribed treatment courses, and 59 (46.1%) abandoned treatment. Of the 128 patients analysed, only 25 (19.5%) could be confirmed to be alive, 65 (50.8%) had died, and 38 (29.7%) had been lost to follow-up, and their status could not be ascertained (Table 2).

### Survival outcomes of the children and adolescents with RMS

**Fig. 3:**
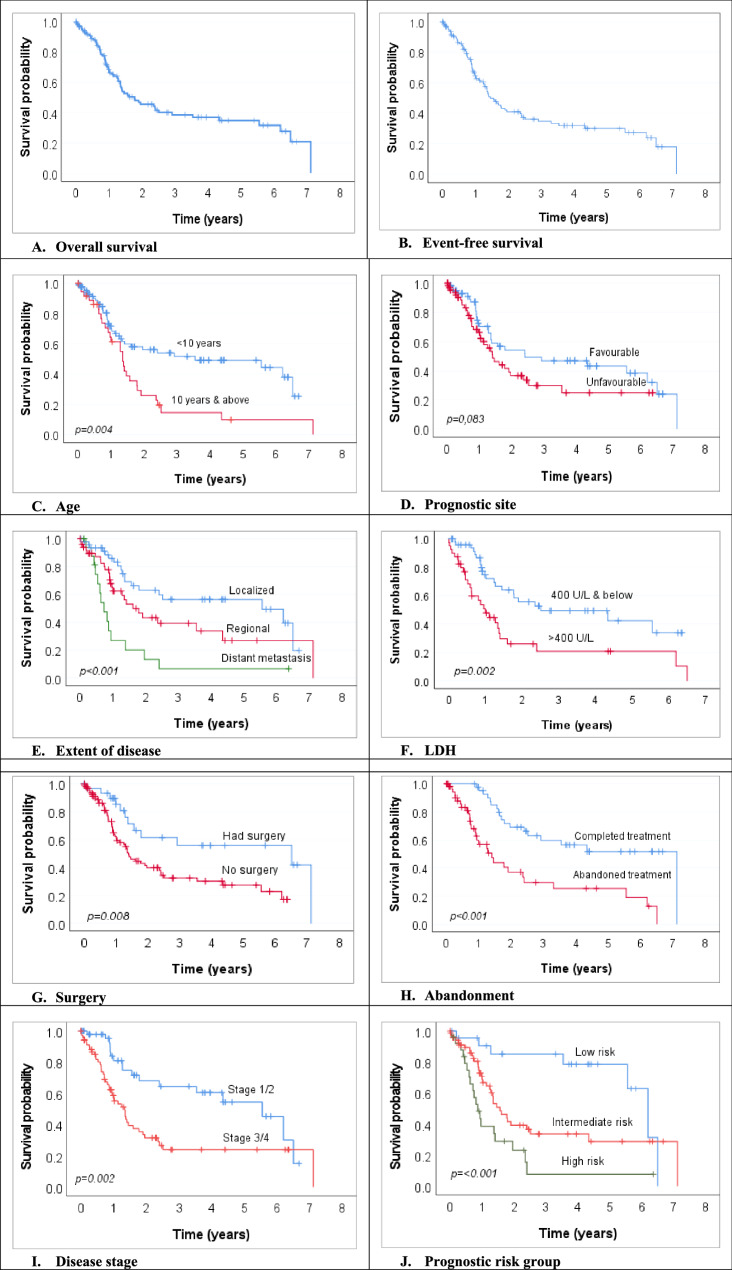
Overall survival curves for children aged < 18 years with rhabdomyosarcoma. **A** Overall survival. **B** Event-free survival. (**C**) Age. (**D**) Prognostic site. (**E**) Extent of disease. (**F**) LDH. (**G**) Surgery. (**H**) Abandonment. (**I**) Disease stage. (**J**) Prognostic risk group

The median follow-up time from diagnosis was 29.1 months (range 2.9–85.4). The median OS was 1.8 years (95% CI 0.9–2.6), while the two- and five-year OS were 45% and 34%, respectively (Fig. 3A). The median EFS was 1.5 years (95% CI 1.1–1.8), while the two- and five-year EFS were 41% and 30%, respectively (Fig. 3B).

Overall survival significantly varied by age: children aged less than 10 years did better compared to those aged 10 years and above (5-year OS 49.1% vs. 9.7%; *p* = 0.004) (Fig. 3C). A significant difference in survival was also seen for prognostic site, disease extent, LDH level, surgical resection, and treatment adherence. Patients with tumours at favourable prognostic sites did relatively better than those with tumours at unfavourable sites (5-year OS 43.2% vs. 24.9%; *p* = 0.083) (Fig. 3D). Patients with localised disease had the best survival outcome compared to those with regional involvement (intermediate outcome) and distant metastasis (worse outcome) (5-year OS 56.4% vs. 27% vs. 6.8%; *p* < 0.001) (Fig. 3E). Likewise, patients with diagnostic LDH of less than 400 U/L had a significantly better survival rate compared to their counterparts with LDH of > 400 U/L (5-year OS 42.4% vs. 20.8%; *p* = 0.002 (Fig. 3F). Surgical resection was associated with a survival advantage over no surgical resection (5-year OS 55.9% vs. 27.5%; *p* = 0.008) (Fig. 3G), and treatment abandonment resulted in a significantly poor survival outcome compared to those who completed treatment (5-year OS 25.4% vs. 51.7%; *p* < 0.001) (Fig. 3H). Survival was also significantly better in patients with lower-stage (stage 1 and 2) disease (*p* = 0.02) (Fig. 3I) and those in the low-risk stratum (*p* < 0.001) (Fig. 3J).

### Univariate Cox proportional hazards model for survival

#### Overall survival (OS)

In the bivariate analysis, age ≥ 10 years at diagnosis (*p* = 0.005, HR = 2.05), parameningeal site (*p* = 0.036, HR = 2.12), “other” primary tumour sites (*p* = 0.033, HR = 3.38), local tumour invasiveness (*p* = 0.001, HR = 2.76), regional nodal involvement (*p* = 0.002, HR = 2.24), distant metastasis (*p* < 0.001, HR = 3.22), IRS stage 3/4 (*p* = 0.002; HR = 2.42), LDH > 400 U/L (*p* = 0.002, HR = 2.42), lack of surgical resection (*p* = 0.010, HR = 2.43), intermediate risk (*p* = 0.015, HR = 2.78) and high risk (*p* < 0.001, HR = 5.58) prognostic groups were significantly associated with poor OS. The OS was also inferior for children with malnutrition (HR = 1.35; *p* = 0.257), unfavourable site (HR = 1.56; *p* = 0.085), tumour size > 5 cm (HR = 1.26; *p* = 0.457), and alveolar histology (HR = 1.38; *p* = 0.301), but these did not reach statistical significance (Table 3).

**Table 3:** Univariate Cox proportional hazards model for Overall survival and Event-free survival (*n*=128)

**Model**	**Overall survival**			**Event-free survival**		
	**HR**	***p*–value**	**95% CI**	**HR**	***p*–value**	**95% CI**
Sex						
Female	Ref.			Ref.		
Male	1.52	0.101	0.92-2.52	1.30	0.271	0.81-2.08
Age (years)						
<10	Ref.			Ref.		
≥10	2.05	0.005*	1.25-3.39	1.78	0.017*	1.12-2.87
Nutrition status						
Normal	Ref.			Ref.		
Malnourished^a^	1.35	0.257	0.80-2.27	1.26	0.360	0.77-2.06
Primary site						
Head and neck– NPM	Ref.			Ref.		
Head and neck– PM	2.12	0.036*	1.05-4.27	1.84	0.064	0.97-3.49
Orbit	1.96	0.092	0.90-4.30	1.70	0.156	0.82-3.51
Genitourinary system	1.16	0.758	0.45-3.00	0.92	0.850	0.36-2.29
Extremity	1.11	0.846	0.40-3.06	0.87	0.784	0.32-2.34
Abdomen	1.96	0.194	0.71-5.41	1.54	0.395	0.57-4.12
Other	3.38	0.033*	1.10-10.37	3.32	0.019*	1.22-9.04
Prognostic site						
Favourable	Ref.			Ref.		
Unfavourable	1.56	0.085	0.94-2.56	1.43	0.141	0.89-2.29
Tumour size						
≤5 cm	Ref.			Ref.		
>5 cm	1.26	0.457	0.69-2.30	1.42	0.236	0.80-2.52
Local tumour extension						
T1	Ref.			Ref.		
T2	2.76	0.001*	1.55-4.91	3.06	<0.001*	1.76-5.31
Nodal involvement						
N0	Ref.			Ref.		
N1	2.24	0.002*	1.35-3.70	2.15	0.002*	1.34-3.44
Metastatic disease						
M0	Ref.			Ref.		
M1	3.22	<0.001*	1.73-5.99	3.06	<0.001*	1.69-5.56
IRS stage						
IRS 1/2	Ref.			Ref.		
IRS 3/4	2.42	0.002*	1.37-4.26	2.00	0.008*	1.20-3.34*
LDH (U/L)						
≤400	Ref.			Ref.		
>400	2.42	0.002*	1.37-4.27	2.30	0.002*	1.35-3.92
Histology						
Embryonal/Botryoid	Ref.			Ref.		
Alveolar	1.38	0.301	0.75-2.54	1.32	0.360	0.73-2.36
Pleomorphic	1.11	0.827	0.43-2.90	1.21	0.670	0.50-2.91
Surgery						
Yes	Ref.			Ref.		
No	2.43	0.010*	1.23-4.79	2.49	0.006*	1.31-4.75
Prognostic risk group						
Low risk	Ref.			Ref.		
Intermediate risk	2.78	0.015	1.22-6.30	20.7	0.045	1.02-4.20
High risk	5.58	<0.001*	2.31-13.50	4.51	<0.001*	2.10-9.70

*IRS* International Rhabdomyosarcoma Study Group (IRSG), *LDH* Lactate Dehydrogenase

*P <0.05; other sites

^a^Both moderate and severe acute malnutrition

#### Event-free survival (EFS)

Likewise, for EFS, the poor prognostic factors in the bivariate analysis were age ≥ 10 years at diagnosis (*p* = 0.017, HR = 1.78), “other” primary tumour sites (*p* = 0.019, HR = 3.32), local tumour invasiveness (*p* < 0.001, HR = 3.06), regional nodal involvement (*p* = 0.002, HR = 2.15), distant metastasis (*p* < 0.001, HR = 3.06), IRS stage 3/4 (*p* = 0.008, HR = 2.00), LDH > 400 U/L (*p* = 0.002, HR = 2.30), lack of surgical resection (*p* = 0.006, HR = 2.49), and intermediate risk (*p* = 0.045, HR = 20.7) and high risk (*p* < 0.001, HR = 4.51) prognostic groups. Likewise, the EFS was also inferior for children with malnutrition (HR = 1.26; *p* = 0.360), an unfavourable site (HR = 1.43; *p* = 0.141), a tumour size > 5 cm (HR = 1.42; *p* = 0.236), and alveolar histology (HR = 1.32; *p* = 0.360), but these did not reach statistical significance (Table 3).

### Multivariate Cox proportional hazards model for survival

#### Overall survival (OS)

In multivariate analysis, the prognostic factors that significantly influenced OS were orbital primary tumour site (*p* = 0.017, HR = 3.73), metastatic disease (*p* = 0.011, HR = 6.45), LDH > 400 U/L (*p* = 0.001, HR = 3.69), lack of surgical resection (*p* = 0.024, HR = 3.50), and intermediate risk prognostic group (0.012, HR = 5.38) (Table 4).

**Table 4:** Multivariate Cox proportional hazards model for Overall survival and Event-free survival (*n*=128)

**Model**	**Overall survival**			**Event-free survival**		
	**HR**	**95% CI**	***p*–value**	**HR**	**95% CI**	***p*** **–value**
Age (years)						
<10	Ref.			Ref.		
≥10	1.38	0.61-3.12	0.433	1.67	0.82-3.43	0.160
Sex						
Female	Ref.			-		
Male	1.01	0.42-2.46	0.979			
Primary site						
Head & neck– NPM	Ref.			Ref.		
Head & neck - PM	0.95	0.34-2.67	0.922	0.91	0.35-2.36	0.847
Orbit	3.73	1.26-11.00	0.017*	2.93	1.06-8.08	0.038*
Genitourinary	1.34	0.39-4.63	0.648	1.07	0.33-3.49	0.907
Extremity	0.77	0.22-2.66	0.677	0.62	0.19-2.07	0.438
Abdomen	0.41	0.10-1.67	0.214	0.30	0.081.18	0.085
Prognostic site						
Favourable	Ref.			Ref.		
Unfavourable	2.47	0.26-23.99	0.435	2.90	0.32-26.18	0.344
Local tumour extension						
T1	Ref.			Ref.		
T2	1.02	0.39-2.68	0.972	1.02	0.40-2.58	0.964
Nodal involvement						
N0	Ref.			Ref.		
N1	1.57	0.70-3.54	0.275	1.38	0.64-2.99	0.414
Metastatic disease						
M0	Ref.			Ref.		
M1	6.45	1.54-26.98	0.011*	6.53		0.006*
IRS stage						
1/2	Ref.			Ref.		
3/4	1.09	0.31-3.84	0.890	0.85	0.27-2.69	0.780
LDH (U/L)						
≤400	Ref.			Ref.		
>400	3.69	1.68-8.12	0.001*	3.44	1.65-7.18	0.001*
Surgery						
Yes	Ref.			Ref.		
No	3.50	1.18-10.40	0.024*	3.58	1.25-10.28	0.018*
Prognostic risk group						
Low risk	Ref.			Ref.		
Intermediate risk	5.38	1.45-19.94	0.012*	3.94	1.32-11.74	0.014*
High risk	2.74	0.49-15.42	0.254	2.22	0.50-9.91	0.296

*IRS* International Rhabdomyosarcoma Study Group (IRSG), *LDH* Lactate Dehydrogenase

*P <0.05

#### Event-free survival (EFS)

Similarly, orbital primary tumour site (*p* = 0.038, HR = 2.93), metastatic disease (*p* = 0.006, HR = 6.53), LDH > 400 U/L (*p* = 0.001, HR = 3.44), lack of surgical resection (*p* = 0.018, HR = 3.58), and intermediate risk group (*p* = 0.014, HR = 3.94) were the prognostic factors significantly associated with poor EFS on multivariate analysis (Table 4).

## Discussion

The current study evaluated the clinical pattern and treatment outcomes among children and adolescents with newly diagnosed RMS in a resource-limited context in Uganda. The study demonstrates generally poor survival outcomes for paediatric RMS in a low-resource setting with high rates of treatment abandonment and low local control rates. Orbital primary sites, metastatic disease, lack of surgical resection, and elevated LDH (> 400 U/L) at the time of diagnosis were found to portend poor survival outcomes, thus identifying potential aspects of management deserving attention for any future efforts for improving RMS survival.

Survival of children with RMS has considerably improved in HICs [12]. A similar trend towards improved survival has been observed in other LMIC settings, as evidenced by findings from Egypt (5-year OS and EFS of 74% and 68%, respectively) [25] and Morocco (10-year OS of 70%) [15]. The observed survival rates in the current study fall short of the trends towards better outcomes as currently seen in HICs– with 5-year OS up to 84.6% and 93.5%, for example, in two European study group reports [26, 27]. However, survival for childhood RMS in most developing countries still remains suboptimal [28]. Our finding is thus concerning, given that the use of a multimodal therapeutic approach has resulted in a reversal of the trend of RMS treatment outcomes in settings, including sub-Saharan Africa [4, 11].

In keeping with international reports [1, 29–33], age at diagnosis was a significant prognosticator among our patient population, with children aged < 10 years demonstrating a survival advantage. Similar better survival among young children compared to adolescents (5-year OS of 71.3% vs. 47.9%) was demonstrated by Yang et al. in an evaluation of 1679 paediatric patients with RMS registered in the SEER database [34]. Three failure-risk categories for age (< 1 year; 1–9 years; >10 years) have long been reported, with significantly worse outcomes among infants and adolescents - setting age as an independent risk factor for treatment failure in RMS [35]. The poor prognosis in infants is thus a result of the reluctance to use intense chemotherapy and aggressive local therapy because of the associated morbidity [36], given that their bone marrow is less tolerant to chemotherapy than older children [1]. This study, however, was unable to estimate the survival outcomes of infants since there were no children under one year.

Primary tumour sites have been suggested to portray epidemiological and prognostic significance, with better survival for tumours in favourable primary sites [1]. However, rather surprisingly, survival in the current study was significantly poor in patients who had primary in the orbit—a favourable site. This is in contrast to the fact that orbital tumours, commonly characterised by embryonal histology, often portend a favourable prognosis [29]. Nonetheless, patients with favourable primary sites in the current study still showed better OS compared to those with primaries at unfavourable sites. The rather incongruous outcome for the orbital site in the current study may be attributed to the challenges of local control. While 10 of the 17 patients with orbital primaries had surgery, only two had complete resection– all with positive margins– and the rest had gross residual disease. Coupled to that, only six of these patients received radiation therapy– the omission of which, according to results from the European Paediatric Soft Tissue Sarcoma Study Group RMS 2005 trial, is associated with reduced overall survival in patients with orbital RMS [27].

The most clinically significant finding in the current study relates to the extent of disease at presentation and the effect on survival outcomes. The OS of patients with metastatic disease and regional lymph node involvement was significantly inferior to those with localised disease. This result is in keeping with the long-established strong prognostic effect of disease extent for patients with RMS, with poor survival not exceeding 30% in patients with metastatic disease [37, 38]. In a report by Badr et al., for instance, over two-thirds (66.7%) of the patients with metastasis at diagnosis died, compared to only about 17.4% of those without metastasis [29]. The prognostic significance of disease extent is modified by tumour histology (embryonal is more favourable than alveolar) and by the number of metastatic sites [38]. In the current study, close to one-half of the metastatic cases had an unfavourable histology and involved multiple (> 3) lesions.

Our study highlights the importance of LDH as a prognostic marker, where elevated LDH (> 400 U/L) conferred a significantly lower OS in children with RMS. Wen-Ling et al. reported similar findings among both children and adults with sinonasal RMS [22], just as a Turkish study by Ustuner et al. among adolescents and adults with RMS [39]. This finding, though less reported in children with RMS, may not be surprising, given the prognostic value of LDH in other childhood cancers, including lymphoma and several solid tumours, including germ cell tumours, neuroblastoma, renal cell carcinoma, and other sarcomas– which is representative of RMS [22, 39]. The prognostic significance of LDH could relate to the importance of LDH as a surrogate for tumour burden and extent of disease. In the current study, the median serum LDH level was higher in patients with a tumour size larger than 5 cm compared to the median level in those with a smaller tumour size. The use of LDH in prognostication, as suggested by the current finding, is of clinical significance, especially in low-resource settings where the capacity for other biological and molecular prognostic markers is lacking. This would be a good surrogate that requires less expertise. However, validation through a large prospective study would be important in investigating if increased LDH values inform possible treatment augmentation. This is already advocated in neuroblastoma to upstage the risk stratification of tumours.

Consistent with the literature [40, 41], there was a statistically significant survival advantage in the current study for patients who had surgical resection over those who did not. Similar improved survival for patients who had surgical resection (5-year OS of 69%) compared to patients who did not have surgery (5-year OS of 47%), irrespective of stage at diagnosis, was reported by Perez et al. [33]. In a Moroccan study, most relapses were attributed to the inability to perform complete resection [15], underscoring the importance of surgical margins. Surgical resection in the current study was, however, low, being pursued in about a quarter (24.2%) of the patients, with complete resection being achieved in only 38.7% of these patients. This compares unfavourably with the 58% rate reported in the European study group– RMS2005 study [26] and attests to the challenges of local control modalities in resource-limited settings [2]. We suppose that the presence of advanced and bulky disease at presentation not amenable to resection could in part explain the observed low surgical resection rate. Likewise, we postulate that lack of surgical services under the same roof at the study site at the time and a high abandonment rate, especially following neoadjuvant chemotherapy with apparent significant cytoreduction giving a false sense of cure to the parents [42], could be the other possible reasons for the low surgical resection rate.

The negative impact of abandonment on overall outcomes in children with cancers has previously been demonstrated [43, 44]. This is reflected in the current study where we observed a high treatment abandonment rate among the children with RMS– a rate higher than the 37% observed in Morocco [15] and South Africa [45] and the 30% in Brazil, South America [46]. Unsurprisingly, overall survival was significantly inferior in those who abandoned treatment compared to those who completed treatment. Abandonment could have therefore possibly contributed to the observed decrease in OS after 5 years among the < 10-year age groups, patients with tumours in favourable primary sites, and those with localised disease. Indeed, though the current study did not explore the reasons for abandonment, factors reported in other studies, like the long duration of RMS treatment necessitating frequent costly hospital travels [45], the false perception by caregivers of the child being “cured” following initial cycles of chemotherapy, and hesitancy to surgical interventions, among other socio-economic factors, may have contributed to abandonment. With only four cancer treatment centres in the whole country, access to cancer treatment services in Uganda becomes not only difficult but economically challenging, with most patient populations having to travel hundreds of kilometres to the treatment centres. Improving access to cancer treatment services through the establishment of more regional and sub-regional cancer treatment centres would therefore warrant attention to lessen the economic burden of accessing treatment.

The limitation of this study lies in the challenge inherent to retrospective studies, such as missing data and records, yet it serves as a motivation to improve clinical records at our institutions and in other similar resource-limited contexts. Likewise, the study was unable to explore the factors contributing to the high treatment abandonment in the study setting, which underscores the need for further prospective study, as this is a major factor contributing to poor treatment outcomes in LMICs. In addition, it was not possible to assess the socioeconomic status of the study participants and its impact on treatment abandonment, poor outcomes, and delayed diagnosis.

## Conclusion

This study has demonstrated that paediatric RMS treatment still poses significant challenges in low-resource settings, with high rates of treatment abandonment and poor survival outcomes. The study also shows that age at diagnosis, extent of disease, primary tumour site, and surgical resection remain important prognostic factors in childhood RMS treatment, just like is the case in HICs. This study therefore casts into focus the wide gap in treatment outcomes of RMS in children and adolescents between high-income countries (HIC) and low- and middle-income countries (LMIC), demonstrating the impact of resource context on the outcome of a reasonably curable disease. The study therefore identifies potential aspects of management deserving attention for any future efforts for improving RMS survival, including the potential prognostic value of LDH, addressing treatment abandonment and improved local control, including surgical resection.

## Supplementary Information

Supplementary Material 1.

Supplementary Material 2.

## Data Availability

The datasets used and/or analysed during the current study are available from the corresponding author on reasonable request.

## Abbreviations

ALP: Alkaline phosphatase
ARMS: Alveolar rhabdomyosarcoma
EFS: Event-free survival
HIC: High-income country
IRSG/IRS: Intergroup rhabdomyosarcoma study group
LDH: Lactate dehydrogenase
LIC: Low-income country
OS: Overall survival
RMS: Rhabdomyosarcoma
UCI: Uganda Cancer Institute
WHO: World Health Organization
