# Supplementary material for: Predictors of survival among children and adolescents with rhabdomyosarcoma treated in a single resource-limited centre —Uganda
Source: BMC Cancer. 2025 Aug 11;25:1299. doi: 10.1186/s12885-025-14735-3 (PMC12337547; doi:10.1186/s12885-025-14735-3)
Supplement: Supplementary file 2 — Supplementary Material 2. [file 12885_2025_14735_MOESM2_ESM.docx]

| Weeks | 1 | 2 | 3 | 4 | 5 | 6 | 7 | 8 | 9 | 10 | 11 | 12 | 13 | 16 | 19 | 22 | 25 | 28 | 31 | 34 | 37 | 40 | 43 | 46 | 49 | 52 |
| --- | --- | --- | --- | --- | --- | --- | --- | --- | --- | --- | --- | --- | --- | --- | --- | --- | --- | --- | --- | --- | --- | --- | --- | --- | --- | --- |
|  | VAI |  |  |  |  |  | VAI |  |  |  |  |  | VAI |  | VAI |  | VAI |  | VAI |  | VAI |  | VAI |  | VAI |  |
|  |  | V | V |  | V | V |  | V | V | V | V | V |  |  |  |  |  |  |  |  |  |  |  |  |  |  |
|  |  |  |  | VDC |  |  |  |  |  |  |  |  |  | VDC |  | VDC |  | VDC |  | VDC |  | VAC |  | VAC |  | VAC |
| Surgery | * |  |  |  |  |  |  |  |  |  |  |  |  |  |  |  |  |  |  |  |  |  |  |  |  |  |
| RT |  |  |  |  |  |  |  |  | * | * | * | * |  |  |  |  |  |  |  |  |  |  |  |  |  |  |
|  |  |  |  |  |  |  |  |  |  |  |  |  |  |  |  |  |  |  |  |  |  |  |  |  |  |  |
| **Drug** | | | |  | **Route** | |  | **Dosage** | | | | | |  | **Day** | | |  |  |  |  |  |  |  |  |  |
| V=Vincristine | | | |  | IV | |  | 2mg/m^2^/day (max 2mg) | | | | | |  | Day 1 | |  |  |  |  |  |  |  |  |  |  |
| A=Actinomycin D | | | |  | IV | |  | 1.25mg/m^2^/day (max 2.5mg) | | | | | |  | Day 1 | |  |  |  |  |  |  |  |  |  |  |
| C-Cyclophosphamide | | | |  | Infusion | | | 1200mg/m^2^/day | | | | | |  | Day 1 | |  |  |  |  |  |  |  |  |  |  |
| D=Doxorubicin | | | |  | Infusion | | | 30mg/m^2^/day | | | | | |  | Day 1, 2 | |  |  |  |  |  |  |  |  |  |  |
| I=Ifosfamide | | | |  | Infusion | | | 1800mg/m^2^/day | | | | | |  | Day 1-5 | |  |  |  |  |  |  |  |  |  |  |
| Mesna | | | |  | Infusion | | | 360mg/m^2^ 3 hourly*5 doses | | | | | |  | Day 1-5 | |  |  |  |  |  |  |  |  |  |  |

**Additional file 2: Treatment protocol for Group 4: All Metastatic Tumours, 2. All Unfavourable Histology, Group 3: Parameningeal Stage 3 and Extremity Stage 3**

*RT=Radiotherapy*
